# Effect of Electrical Stimulation on Ocular Cells: A Means for Improving Ocular Tissue Engineering and Treatments of Eye Diseases

**DOI:** 10.1155/2021/6548554

**Published:** 2021-11-17

**Authors:** Fatemeh Sanie-Jahromi, Ali Azizi, Sahar Shariat, Mohammadkarim Johari

**Affiliations:** Poostchi Ophthalmology Research Center, Department of Ophthalmology, School of Medicine, Shiraz University of Medical Sciences, Shiraz, Iran

## Abstract

Tissue engineering is biomedical engineering that uses suitable biochemical and physicochemical factors to assemble functional constructs that restore or improve damaged tissues. Recently, cell therapies as a subset of tissue engineering have been very promising in the treatment of ocular diseases. One of the most important biophysical factors to make this happen is noninvasive electrical stimulation (ES) to target ocular cells that may preserve vision in multiple retinal and optic nerve diseases. The science of cellular and biophysical interactions is very exciting in regenerative medicine now. Although the exact effect of ES on cells is unknown, multiple mechanisms are considered to underlie the effects of ES, including increased production of neurotrophic agents, improved cell migration, and inhibition of proinflammatory cytokines and cellular apoptosis. In this review, we highlighted the effects of ES on ocular cells, especially on the corneal, retinal, and optic nerve cells. Initially, we summarized the current literature on the in vitro and in vivo effects of ES on ocular cells and then we provided the clinical studies describing the effect of ES on ocular complications. For each area, we used some of the most impactful articles to show the important concepts and results that advanced the state of these interactions. We conclude with reflections on emerging new areas and perspectives for future development in this field.

## 1. Introduction

The cell therapy strategy is rapidly growing for use in the treatment of degenerative ocular diseases [1]. There are numerous studies on the use of stem cell therapy for the treatment of ocular and retinal degenerative diseases [2–4]. Clinical application of stem and progenitor ocular cells has become achievable nowadays. Embryonic stem cells have benefited Stargardt's macular dystrophy and dry age-related macular and retinitis pigmentosa patients [5]. Optic nerve damaged due to trauma, glaucoma, demyelinating optic neuropathy or toxic optic neuropathy, retinitis pigmentosa (RP), age-related macular degeneration (AMD), and retinal vascular accident-related diseases are the most common retinal diseases that are expected to profit from beneficial effects of stem cell therapy in the near future [6]. Corneal epithelial progenitor cell therapy has also demonstrated a promising approach for corneal regeneration in limbal stem cell deficiency (LSCD) [7]. The advancement of cell therapies for ocular disorders is due to the ideality of the eye for cell-based therapy [8]. These include easy surgical access, the possibility of a thorough examination of the transplanted tissue after cell therapy, and the relative immunological privilege of the eye. The success of cell therapy depends on the use of appropriate tissue engineering methods for cell processing and the use of biophysical and biochemical agents to stimulate cells to proliferate, migrate, and settle in the exact loci [9]. One of the physical factors that have been considered to stimulate cells is electrical stimulation (ES). ES is a therapeutic technique that uses low-intensity electric current and has successful results in the treatment of neurodegenerative diseases [10, 11]. This process does not involve any generation of perceived heat. Instead, the nerve endings are stimulated directly for therapeutic effects [12, 13]. Over the past two decades, the use of ES for eye treatment has been highlighted due to the favorable results in animal models and human experiments. Luu et al. have recently reviewed the history of electrotherapeutics in ophthalmology and introduced the novel ophthalmic instrumentation [14]. The eye is an electric-based organ and the ocular cells, particularly in the retina, use endogenous electrical currents to function [15]. So it seems that ES is a proper biophysical stimulating factor to be used in tissue engineering to alter cell behavior [16]. From the cellular point of view, the cell uses electrical mechanisms to establish and regulate its biological processes including proliferation, migration, and nerve stimulation [17]. Recent studies have shown that ES can increase cell survival, migration of transplanted cells, and improve the formation of axonal regeneration and synapse function [18]. In recent years, the synergy between cell therapy and ES has been considered as a potential candidate for the treatment of degenerative diseases [19]. This study is aimed at reviewing the recent investigations on the effect of ES on ocular cells that could improve corneal and retinal cell behavior for ocular tissue regeneration and treatment.

## 2. The Effect of ES on Stem Cells

One of the characteristics of stem cells is their ability to be differentiated into various cell types based on their microenvironment including the biochemical and biophysical stimuli applied to them [20, 21]. Electrical stimulation seems to be a promising tool for modifying stem cell behavior. It has been shown that vascular endothelial progenitor cells were affected by a direct current of electric field signals, and their migration was guided through the establishment of the vascular endothelial growth factor (VEGF) receptor signaling cascade *in vitro*. This study confirmed the role of ES in the control of endothelial cell migration that is important both in the wound healing process and tissue engineering [22]. Examination of murine adipose-derived stromal cells (mASCs) in direct current (DC) fields showed that the mASCs migrated to the cathode in the electric field and were arranged perpendicular to the field vector. It was also shown that ES caused a transient increase in cytosolic calcium [23]. The analysis of migration in human neural stem cells treated with DC electrical fields (EFs) also confirmed the directional migration of neural stem cells toward the cathode in a time and voltage-dependent manner. This study showed that neural stem cell migration was not dependent on Rho/ROCK and SDF-1/CXCR-4 signaling pathway [24]. The migration of Schwann cells in the electric field was surveyed in another study, and it was shown that Schwann cells migrate toward the anode in the electric field. This study provided a comprehensive gene analysis that used next-generation RNA sequencing to evaluate the genes and signaling pathways that were involved in EF-stimulated cell migration. According to the results, 21 downregulated and 10 upregulated pathways were identified in the control of ES-induced cell migration. The cascades had a role in controlling the actin cytoskeleton, focal adhesion, and PI3K-Akt pathways [25]. This study and other similar studies showed that ES could affect cellular activities and behavior of stem cells such as division, migration, and cell death by initiating intracellular molecular pathways. Among the tissue sources for stem cell extraction that have always been of interest to researchers are ocular tissues such as the cornea, retina, and ciliary bodies. Ocular cells have distinct electrical properties that might be helpful for the invention of novel ES-based therapeutic applications and methods of tissue engineering. In a study by Klyce, it was demonstrated that corneal epithelial cells have a distinct electrical profile, membrane potential, and membrane resistance that are based on the ion diffusion around cell membrane [26]. Moreover, it was shown that electric fields in the vicinity of corneal epithelial wounds are different from normal corneal tissues [27] that are indicative of the role of ES in the process of corneal wound healing [28–30]. The role of calcium and potassium in establishing electrical currents in the lens cells was also confirmed previously [31]. ES exerts galvanotropic effect on RGC axon growth and provides a helpful means for cell therapy of optic nerve degenerations [32]. Due to the electrical features of ocular cells, it seems that ES can be suitably applied for modification of the ocular cell behavior.

## 3. The Effect of ES on the Ocular Cell In Vitro and In Vivo

Due to its ability for noninvasive penetration to the cell, ES seems to be a promising candidate both as a therapeutic tool and an aid to tissue engineering. Various studies have shown the effect of ES on cells, tissues, and biological processes including embryogenesis, wound healing, and repair [23], as well as on cell migration, DNA synthesis, DNA repair, and gene expression [33–35]. Different issues were under the study of tissue engineers, among them are stimulation of cell proliferation and direction of cell migration [36–38]. ES is a cost-effective and durable stimulating factor and is easy to reproduce. ES has been shown to relieve pain, strengthen blood circulation, and have unparalleled clinical effects in the treatment of disease [39]. Also, many studies are available that report the effect of ES on the manipulation of cellular behaviors *in vitro* and *in vivo* [40, 41]. ES stimulates cells to initiate intracellular signaling pathways, thereby leading to direct cell activity and function [16, 42]. There are numerous reports on the effect of ES on ocular cells, especially on the corneal and retinal cells. Hu et al. have recently demonstrated that ES could significantly promote the proliferation activity of dorsal root ganglion cells (DRGs) and inhibited cell apoptosis. The results of this study also showed that ES could activate the Wnt/*β*-catenin signaling pathway and exert a protective effect on the injured DRGs [43]. Recently, it has been reported that ES increases the expression of mRNA of brain-derived neurotrophic factor (BDNF) and consequently its protein level in the Müller cell *in vitro* [44]. Also, it is reported that ES can increase the number of surviving retinal ganglion cells (RGCs) *in vivo* due to the increased level of insulin-like growth factor-1 (IGF-1) produced by Müller cells [45]. Increased expression of ciliary nerve trophic factor (CNTF), B-cell lymphoma 2 (Bcl-2) expression [46], and fibroblast growth factor 2 (FGF2) [46, 47] after ES treatment in cultured retinal Müller cells has also been reported. Type L calcium channels are shown to have a role in the upregulation of FGF2 expression [44, 48].  Table 1 provides a brief description of the *in vitro* and *in vivo* studies focusing on the effect of ES on the intracellular mechanisms of retinal and corneal cells (see references (44–59)). As demonstrated in Table 1, an investigation of 16 *in vitro* and *in vivo* studies revealed ES as a role player in cell migration. The migration was directional, i.e., toward the cathode pole of the electric field. The ES-induced migration is then hypothesized to accord with a similar electrical mechanism *in vivo* which conveys healing cells toward the defected ocular area. In addition, ES upregulates some intrinsic factors comprise of pSrc, bFGF, BDNF, IGF-1, BCL-2, and CNTF in ocular cells. These pathways altogether stimulate cell proliferation and the expression of progenitor cell markers which lead to advanced activity of retinal ganglion cells (mostly Müller cells). This phenomenon brings ES a regenerative potential and delay ability to hamper photoreceptor degeneration. The efficacy of ES was reported to depend on the electric field strength and the cooperation of medium ingredients for instance growth factors and matrix metalloproteinases (MMPs). All in all, the studies suggested ES be a potentially safe and beneficial trend for *in vitro* tissue engineering application as well as and *in vivo* healing procedures.

## 4. The Most Recent Clinical Studies on the Effect of ES on Ocular Diseases

A review of existing studies shows that ES is a noninvasive tool for the treatment of some retinal and optic nerve diseases. There are several strategies for applying ES; including stimulation through the cornea, transorbital, and transpalpebral, also stimulations by implantations of the retinal prosthesis through subretinal, epiretinal, and transchoroidal pathways, as well as the direct stimulation of the optic nerve or brain. The most important mechanisms of action of ES seem to be the increased production of neurotrophic factors, improved blood circulation, and inhibition of proinflammatory cytokines [60]. The first report of the use of ES in the eye was made in 1755 (by Charles LeRoy) for a blind patient with cataracts [61]. Since then, many different studies have been performed on animal models and clinical trials that have yielded promising results (see below). The researchers have shown that subthreshold currents could not only improve vision in retinitis pigmentosa (RP) patients but also induce neuroprotective factors by inducing expression [62]. These studies led to an interest in research on the effects of ES in animal experiments and later in clinical trials for the treatment of blindness. Different animal models of mice, cats, and rabbits have been studied to evaluate the optimal effects of ES on the retina and optic nerve [63–66], and promising outcomes were reported such as increased survival of RGCs in rats [45] and increased expression of insulin-like growth factor-1 (IGF-1) in Müller and inner retinal cells [67]. It was also shown that repeated stimulation of ES had a greater effect on improving optic nerve function [67]. At the time of writing, twenty-two ES-based clinical trials for eye disease have been registered at http://clintrials.gov/. According to the details provided by http://clintrials.gov/, in these studies, different methods have been used to apply ES to the eye. Most of these experiments are still ongoing, and the results published so far have been promising.  Table 2 shows a list of ongoing studies in this area. Below, we have provided a brief description of the completed and recruiting clinical trials on ES application for the treatment of ocular complications.

*NCT01282827*: in this study, Sabel et al. investigated the visual function recovery in optic nerve damaged individuals by applying a noninvasive transorbital alternating current stimulation (ACS). Their double-blind study comprised 22 patients (12 treated with ACS and 10 stimulated with placebo). ACS applied 40 minutes each episode for ten days. Visual outcomes and EEG (electroencephalogram) were measured before and after the procedure. The results demonstrated significant improvement of a visual field (VF) detection deficit and visual acuity (VA). The alpha-band changes in the EEG indicated increased synchronization in posterior brain regions. Patients' visual improvements were reported stable for almost two months [68].

*NCT00804102*: this study assessed transcorneal electrical stimulation (TES) safety and its effect on the visual function of RP patients. In this work, 24 patients were treated by TES (5 ms biphasic pulses; 20 Hz; DTL electrodes, 30 minutes per week for six consecutive weeks). Individuals underwent either sham or TES. Based on the study, VA, VF, electroretinography, dark-adaptation (DA), color discrimination, and electrical phosphine threshold (EPT) were assessed four times. Results showed the safety of TES for RP patients. VF area and scotopic b-wave amplitude were reached statistically significant. However, a larger sample size was needed for further definition of optimal stimulation parameters [69].

*NCT01280877*: this study was run to assess the effect of repetitive transorbital alternation current stimulation (rtACS) in partially blind individuals with optic neuropathy. Forty-five patients were treated with rtACS and 37 patients were stimulated with the Sham procedure. Daily setting applied for 50 min in a duration of 10 weekdays. Study measurements include primary outcome (super-threshold visual fields 48 hrs after the last treatment day and two months follow-up) and secondary outcome (near-threshold visual fields, reaction time, VA, and resting-state EEGs). VF improved in the rtACS-treated patients. EEG revealed signs of neuromodulation which was suggested as probable brain plasticity modulation. In this work, rtACS was reported as a safe and effective therapy for restoring vision [70].

*NCT01835002*: safety assessment of TES application was investigated in Jolly et al.'s work. TES was induced in a total of 105 patients with RP. Weekly TES was applied for six months on one eye, followed by another six months of no stimulation. Outcome measurements include safety (frequency and severity of adverse events), intraocular pressure (IOP), and central retinal thickness. The study reported transient dry eye symptoms and no other serious adverse events post TES. The safety profile of TES was then confirmed [71].

*NCT01847365*: Wagner et al. demonstrated the role of TES in a total of 14 patients with RP. In this trial, weekly TES was performed for six months followed by another six months of observation. Patients were examined every other 3 months. Primary outcome measurements were TES safety and adverse effects. Secondary outcomes are explored as the measurement of structural and functional efficacy. This study showed the safety and well tolerance of TES application. However, no significant difference in VF improvement was reported [72].

*NCT02086890*: this clinical trial was started in 2014, and it is still active. There are three articles published so far information. Though, the final investigations are yet to come. Bittner and Seger assessed the TES effects on RP subjects. In this small-scale study, at first, four of seven patients demonstrated improved VA, quick contrast sensitivity function (qCSF), and VF post-TES. TES was induced weekly for a total of six weeks. The second step was monitoring three of these well-responded participants longitudinally. The aim was the investigation of potential visual function declination due to the natural RP process. The duration of their responses and administered retreatments was then determined. These participants received three to six TES courses over 29-35 months. Each course included six weekly sessions. These episodes occurred every four to 16 months in this prospective work. The results showed significant improvement in VA and VF and no significant decline for VA and qCSF from baseline. Altogether, this study suggested TES as prevention for slowly diminishing vision in RP patients. The authors indicated a larger scale of study for the confirmation [73]. In another report, post-TES changes in visual function and ocular and retinal blood flow (RBF) were investigated among RP patients. Twenty-one participants randomly received TES or electro-acupuncture for half-hour sessions during six weeks. Control group treated with inactive laser acupuncture as sham-operated. The outcome measurements were VA, qCSF, VF, AdaptDx scotopic sensitivity, spectral flow, color Doppler imaging of the central retinal artery (CRA), and RBF in macular capillaries pre and post the study. Results showed significant improvement in retrobulbar CRA mean flow velocity, RBF, and VA in both electrically stimulated groups. In summary, this report recommended electro-stimulation as an inducer factor for retinal blood flow and visual function improvements in RP patients [74]. The third study was conducted to investigate whether reduced blood flow in the retrobulbar CRA is related to the degree of visual function loss in RP patients. Eighteen participants were assessed with VA, qCSF, VF, and dark-adaptation with the AdaptDx; multifocal electroretinography (mfERG), CRA peak systolic velocity (PSV) and end-diastolic velocity (EDV), and mean flow velocity (MFV). Each eye was examined twice in two visits within a month. The outcome confirmed MFV changes do affect visual function suggesting the application of therapies that have such indirect impact [75].

There are additional 16 clinical trial studies which either still recruiting or did not have published results. The aim of their studies and excised methodologies and also primary outcomes are reported below.

*NCT01600300*: this randomized, quadruple-blinded trial is completed; however, the results have not been published yet. The aim was to evaluate the effect of external ES on the VA of patients with AMD. Forty subjects were involved in this trial receiving either ES with Tesmac device twice daily for 10 days or sham-operated. V/A changes were the outcome measurements.

*NCT01630291*: this trial is completed with no published results found. This nonrandomized study included four patients with severe dry eye. The purpose was to evaluate the effect of ES of the tear gland-related nerve on tear production. Measurements were assessed by the Schirmer test.

*NCT02019927*: this trial is completed with no published results found. The study design was randomized double-blinded and was to evaluate the effect of TES on visual function. Ninety-seven participants were included: patients with ocular trauma, patients with optic neuritis associated with MS, patients with nonarteritic anterior ischemic optic neuropathy (NAAION), and sham-operated groups. V/A, VF, IOP, and OCTs were assessed during the process.

*NCT04008589*: this randomized, controlled trial is completed with no published results found. The aim was to evaluate the effects of ES of the brain on visual functions in patients with stroke-related field deficits. These forty-five patients were divided into either ES or sham-operated groups. The outcome measurements were assessed by visual field perimetry and EEG.

*NCT02405143*: This work's recruitment status is unknown. Fifty individuals with occipital stroke-related hemianopia underwent either transcranial electrical stimulation or sham stimulation. The purpose was to evaluate the ES effects on VF improvements. The study was run randomized, double-blind, and the high-resolution perimetry was used for outcome measurements.

*NCT02495935*: this randomized, triple-blinded trial is still in progress. The purpose of this prospective study was to evaluate applied TES on visual functions of adult patients with amblyopia. Forty subjects were treated either with TES or Sham TES. The treatment was 30 minutes of weekly TES (20 Hz) for twelve weeks. The same intervals were undergone for the sham group with no ES application. The outcome measurements were V/A changes pre and post the trial.

*NCT02699216*: this study is withdrawn. The aim was to evaluate the effectiveness of transpalpebral microcurrent ES on the visual function of dry macular degeneration patients. The ES set was 5 Hz to 80 Hz during each spot (40 sec), 200 micros A voltage. V/A and microperimetry were the outcome measurements.

*NCT03239418*: This study's recruitment status is unknown. The trial was randomized and triple blinded. The purpose was to investigate the impact of neuromuscular electrical stimulation (NMES) on eye opening and closure in patients with blepharoptosis or lagophthalmos (CN III or CN VII palsy). Sixty participants were divided into NMES treated groups or sham-operated. NMES protocol was 30 min per day for five days with the same intervals for the sham group. Investigation of eyelid function was the outcome measurement.

*NCT02548572*: this study is withdrawn as they could not recruit subjects. The aim was to investigate the effect of TES on the visual deterioration of RP patients. Participants were supposed to undergo weekly TES for one year.

*NCT04042363*: this study is still in progress. It was designed as a randomized, interventional, controlled, and blinded trial which targets the effect of transorbital electrical stimulation on nerve remyelination and neuroprotection. The study participants had MS with an acute episode of retrobulbar optic neuritis. Forty-five patients were recruited including TES and sham-operated group. The TES/sham intervention was applied in 10 sessions during two consecutive weeks and a total of 14 visits.

*NCT04010994*: This study is enrolling by invitation. Fifty glaucoma patients were included in this study. The aim was to evaluate the effect of ES on VF as a home treatment. All the patients had optical neuropathy and underwent TES using 8-12 Hz with 0.5-1.5 mA intensity, during the first two weeks 1/day, after 2/week for 10 weeks. In addition, further interfering factors were assessed: the role of mental stress (or stress resilience), the status of biomarkers, such as the systemic stress hormone levels and blood supply to the eye and brain (specifically vascular dysregulation), and the influence of personality, anxiety, depression, and lifestyle. Perimetry, EEG, and dynamic vessel analysis were used for outcome measurements.

*NCT04017234*: this randomized, single-blinded trial is still recruiting. The aim was to assess the effect of TES on the prevention of myopia progression (PMP). Twenty-seven elementary school students will be treated eye exercise, and TES as an experimental group or only will receive health education as a controlled group. The treatment frequency will be three times per week for four weeks. The outcome measurement will be eyeball diopter detection.

*NCT02540148*: this randomized, single-blinded study is still recruiting. Sixty individuals with dry age-related macular degeneration (AMD) will be divided into two groups of either receiving ES or sham-operated. The aim is to evaluate the impact of microcurrent ES on the VA of the subjects. ES will be applied in weeks 1, 2, 14, and 26 with the Nova Oculus device. The control group will be treated with the same intervals by a nonfunctional device. V/A will be assessed pre and post the trial.

*NCT04634383*: this trial is still recruiting. The purpose was to introduce artificial vision in participants with blindness by wireless ES implanted in their visual cortex. The responses of patients to the ES were assessed. Five patients will take brain surgery and have the wireless floating microelectrode arrays (WFMAs). ES will then be induced, and visual percepts will be assessed. The testing will occur weekly in a one to three-year period.

*NCT04648085*: this study is in progress to date. Forty-two individuals with dry eye were treated by TES for 20 minutes at a frequency of 100 Hertz (Hz). This prospective, single-arm pilot study was aimed at assessing the effect of TES on corneal nerves and chronic ocular pain.

## 5. The Effect of ES on Ocular Function in the Real World

Recently, ES has been investigated for treating multiple ophthalmological and neurologic diseases that apply a low current (range of *μ*A) stimulus to activate injured tissues and nerves. Glaucoma, AMD, retinal vasculopathy, such as diabetic retinopathy or retinal vascular obstructions, and RP are leading causes of irreversible blindness in the developed world. The studies show that patients with RP demonstrate significant improvements in color discrimination, visual field area, and scotopic ERG parameters [69, 76, 77]. AMD is another eye disease that affects a person especially in old age and causes the loss of photoreceptor cells in the macula and eventually blindness. In patients with AMD, ES was shown to improve both visual function and best-corrected visual acuity in both wet and dry AMD, although it was more effective in dry type than wet type [78–80]. ES was found to improve both VA and VF in some patients with central or branch retinal artery obstruction. Multifocal ERGs demonstrated that the implicit time and amplitudes of all waves significantly improved after the ES treatment, suggesting that ES impressed favorable effects on both the inner and outer retinal neurons in these patients [81–83].

Glaucoma is a progressive optic neuropathy that is due to increased IOP and results in RGCs lost with concomitant VF limitation; ES exhibited improvement in glaucomatous VF defects and effective hypotensive response in subjects with glaucoma [84, 85]. Prospective dimensions of this technology in the future should be considered the availability of the mobile instruments for whole patients with the less invasive and easier to apply method. As it has been proved that eye electrical stimulation is a safe method, it is suggested that more developed and distinguished devices could lead the teleophthalmology therapies. Patients may benefit from adjustable eye pads whose ES dosage and duration would be regulated or prescribed remotely by an ophthalmologist. This procedure reduces patients' referrals to the eye clinics, and it is a time and cost-benefit matter. However, the safety and indication of such devices should be investigated more precisely. The value ES promises would be improvements in visual fields, optic nerve, and retinal revival and as a hole improving vision and sight in the aforesaid patients.

## 6. Conclusion

ES has provided tangible assistance not only to manipulate ocular cell behavior but also to individuals with ocular impairments for decades. This technology has allowed injured cells to react and restore their functions in the neuro-ophthalmologic pathway. The amount of evidence for restoring visual function in various diseases using ES is rapidly growing. Today, ES is an important tool available both to clinicians working in the field of neuro-ophthalmology rehabilitation and researchers working in the field of ocular tissue engineering. It is expected that the prevalence of ES will likely increase in the next decades with an increase in the aging population, age is the main risk factor for the development of several eye diseases such as AMD, retinal vascular disease, and glaucoma, so with these changes in population demographic states, rehabilitation with ES technology may highlight its role in future. A new understanding of the specific stimulation settings provides an opportunity to see further increases in the efficacy of this intervention.

## Figures and Tables

**Table 1:** A brief description of the *in vitro* and *in vivo* studies on the effect of ES on the ocular cells.

Source	Cell	ES pattern	Outcome	Study type	Ref
Rat	MC	Biphasic pulses, duration, 1 ms; frequency, 20 Hz; current, 10 mA, for 30 min.	(i) Upregulation of total BDNF (increase in intracellular but not in extracellular amounts) (ii) Induction was dependent on calcium influx through L-VDCCs (iii) The ES of MC may be applicable for BDNF demands of the retina.	In vitro	[30]
	RGCs MC	TCES, biphasic current pulses 100 *μ*A, 20 Hz, 0 to 3 ms/phase for 1 h.	(i) Upregulation of IGF-1 (ii) IGF-1 appearance was first recognized in the endfeet of MC and then distributed into the inner retina. (iii) The degree of rescue depended on the strength of the electric charge. (iv) TCES revives the RCGs by increasing IGF-1 levels by MC.	In vivo	[31]
	Retinal cell MC	TCES, 300 *μ*A, 20 Hz, 3 ms/phase for \ 1 h, every 3 d after exposure to light for up to 14 d.	(i) Upregulation of Bcl-2, CNTF, and BDNF. (ii) Downregulation of Bax. (iii) Upregulation of Bcl-2 and CTNF in MC. (iv) The rate of reviving depended on the strength of the electric charge. (v) TCES activates the intrinsic survival system by upregulation of antiapoptotic genes and downregulation of proapoptotic genes. Thus, prevents or delays photoreceptor degeneration.	In vivo	[32]
	MC	1 ms pulse (20 Hz, 0–10 mA), continuous treatment for 30 min	(i) Upregulation of FGF-2 messenger RNA and protein. (ii) ES upregulates the production of FGF-2 in retinal Mueller cells.	In vitro	[33]
	MC	Biphasic pulses, duration, 1 ms; frequency, 20 Hz; current, 0–10 mA, for 30 min.	(i) Upregulation of IGF-1 transcription through the function of L-type Ca2+ channels and Ca2+ influx. (ii) Downregulation of IGF-1 protein production. (iii) TES induces IGF-1 production in cultured MC.	In vitro	[34]
	MGC MC	3 ms biphasic pulses, 20 Hz, 300–1600 *μ*A, continuous treatment for 1 h	(i) Decrease activated microglia cells with ameboid shapes. (ii) Increased reactive MC. (iii) Inhibits IL-1*β* and TNF-*α* in microglia cells. (iv) Positive regulative effect on the production of BDNF and CNTF in MC. (v) ES is considered for delaying the progression of photoreceptor degeneration.	In vitro	[35]

Murine	Rho−/− MC	One or two sessions of trans-palpebral ES or sham treatments for 7 consecutive days (4 spots on eyelids, 100 *μ*A for 40 s)	(i) ES directly stimulated cell proliferation and the expression of progenitor cell markers in MC cultures, via bFGF signaling. (ii) ES might have a regenerative potential by releasing bFGF release and inducing MC proliferation and progenitor cell properties.	In vivo and in vitro	[36]
	RPC (postnatal day 1, green fluorescent protein+)	100 *μ*A pulse, (5 s duration, 1/min for 4 days)	(i) Higher levels of the early photoreceptor marker CRX and PKC (ii) Significantly lower levels of GFAP. (iii) Pronounced neuronal morphologies with significantly longer dendritic processes and larger cell bodies. (iv) ES has a possible role in directing progenitor cells toward differentiation.	In vitro	[37]
	RPC (postnatal day 1, green fluorescent protein+)	Monophasic 5 V square-wave pulses (1 ms duration for 100 ms, 3 s bursts, 1/min, for 3 days)	(i) Lower levels of N-cadherin. (ii) Comparable levels of Cdc42. (iii) Higher levels of ßIII-tubulin. (iv) Oscillating calcium influxes (v) Increasing neural differentiation (vi) Activity-dependent dendritic morphogenesis toward early functional morphology. (vii) ES directed RPCs toward developing functional properties.	In vitro	[38]
	CEC (Pax6+/− vs. Pax6+/+)	200 mV/mm for 2 h	(i) Pax6+/+ cells: cathodally migration. Upregulation of cathodal pSrc activity and total levels. (ii) Pax6+/− cells: response to ES in speed and displacement changes but no significant in direction variation. No changes with pSrc. (iii) ES causes cell migration and it depends on the level of Src signaling.	In vitro	[39]

Rabbit	CEC	Transcutaneous ES (continuous DC electric field, 100 mV/mm, 30 min)	(i) TCES elevated the rate of corneal healing mostly in the first 24 h	In vivo	[40]
	CEC CSF	-4 V/cm for CEC -6 V/cm for CSF For a duration of 20, 50 min, 4 h	(i) After 20 min (ii) Formation of spindle-shaped cells (iii) Galvanotropism (iv) After 50 min (v) Migration of epithelial cells toward the cathode. (vi) Migration of stromal fibroblasts toward the anode. (vii) After 4 h (viii) Treatment with ES > 10 V/cm caused cellular damage. (ix) ES is responsible for corneal cell migration.	In vitro	[41]

Bovine	CEC	100-250 mV/mm for 5 h.	(i) 10% FBS medium: significant galvanotropism, reorienting toward electric field vector, threshold ES < 100 mV/mm. (ii) FBS-free medium: no reorientation occurred until 250 mV/mm. (iii) FBS-free medium + EGF, bFGF, or TGF-b1 (singly or in combination): significant cathodal galvanotropism at low field strengths. (iv) Combination of growth factors and ES increases cell migration.	In vitro	[42]

Human	CEC	0–150 mV/mm for 3 h at 37°C	(i) Upregulation of MMP3. (ii) ES benefits from MMPs for applying on cell migration. (iii) ES improved directional (cathodal) collective cell migration.	In vitro	[43]
	CEC	100 mV/mm DC field, the current remains below 0.6 mA, at 37°C, for 1 h	(i) Directed (cathodal) migration of CECs. (ii) This migration is similar to keratinocytes, but faster. (iii) ES plays an important role in human corneal wound healing.	In vitro	[44]
	Primary vs. transformed CEC	100–250 mV/mm, 2-5 h	(i) Both types had cathodal directed migration (ii) Reorientation and directional migrations were voltage and serum dependent. (iii) EGF facilitates cell migration. (iv) Transformed human cells needed higher ESs (250 mV/mm) to show guided migration. (v) ES might affect cell morphologies and directed cell migration in the healing process.	In vitro	[45]

A: ampere; Bax: Bcl-2-associated X; Bcl-2: B-cell lymphoma 2; BDNF: brain-derived neurotrophic factor; Cdc42: cell division control protein 42 homolog; CEC: corneal epithelial cell; CNTF: ciliary neurotrophic factor; CRX: cone-rod homeobox; CSF: corneal stromal fibroblasts; EF: electric field; EGF: epidermal growth factor; ES: electrical stimulation; FBS: fetal bovine serum; FGF: fibroblast growth factor; GFAP: glial fibrillary acidic protein; h: hour; IGF: insulin-like growth factor; IL: interleukin; L-VDCCs: L-type voltage-dependent calcium channels; MC: Müller cell; MGC: microglia cell; min: minute; MMP: matrix metalloproteinases; Pax6: paired box protein; PKC: protein kinase-C; RGC: retinal ganglion cell; RPC: retinal progenitor cell; s: second; TCES: transcorneal electrical stimulation; TNF-*α*: tumour necrosis factor alpha; V: volt.

**Table 2:** A list of clinical trials on the effect of ES on ocular diseases.

NCT number	Conditions understudy	Interventions	*N* (age)	Outcome	*L*	Status	Start date
NCT01600300	MD	Two period treatment with Tesmac device, twice/day for 5 days, with a two-day interval	40 (≥50)	NA	NA	Completed	2002

NCT01282827	Visual impairment	Repeated tACS (2 to 9 pulses, <1000 *μ*A)	40 (18-75)	(i) Significant improvement of a VF detection deficit (ii) Significant improvement of VA (iii) EEG: increased synchronization in posterior brain regions (iv) Patients' visual improvements were stable for almost two months	Germany	Completed	2006

NCT00804102	RP, macula off, POAG, hereditary MD, treated retina detachment, retinal artery occlusion, retinal vein occlusion, nonarthritic-anterior-ischemic optic-neuropathy, hereditary autosomal dominant optic atrophy, dry AMD, ischemic ME	Transcorneal ES (Dawson-Trick-Litzkow electrode attached to patient eye)	80 (≥18)	(i) Safety of TES for RP patients. (ii) VF area and scotopic b-wave amplitude were reached statistically significant (iii) A larger sample size was needed for further investigations	Germany	Completed	2008

NCT01280877	Optic nerve diseases Optic nerve injuries Optic neuropathies	tACS (8 to 14 pulses, <1000 *μ*A), 1 pulse per minute during 25-35 min	90 (≥18)	(i) VF improved in the rtACS-treated patients. (ii) EEG: signs of neuromodulation (suggested as probable brain plasticity modulation) (iii) rtACS was reported as a safe and effective therapy for restoring vision	Germany	Completed	2010

NCT01630291	Dry eye	Electrode device for lacrimal gland stimulation	4 (≥18)	NA	Mexico	Completed	2012

NCT01835002	RP	Weekly ES using Okustim® device for a period of 30 min for 6 months	105 (18-80)	(i) Transient dry eye symptoms (ii) No other serious adverse events post TES. (iii) The safety profile of TES	Germany, UK, the Netherlands, Italy, Denmark, and Norway	Completed	2012

NCT01847365	RP	Weakly transcorneal ES using Okustim® device for a period of 30 min for 6 months	14 (≥18)	(i) Safety and well tolerance of TES application (ii) No significant difference in visual function improvement	United Kingdom	Completed	2013

NCT02019927	NAION, trauma, MS	Transcorneal ES using the OkuVision device	97 (≥18)	NA	United States	Completed	2013

NCT04008589	Stroke	Repetitive transorbital AC stimulation combination of transcranial direct current stimulation and rtACS	45 (18-75)	NA	Germany	Completed	2014

NCT02086890	RP	Electro-acupuncture Laser acupuncture transcorneal ES	21 (≥18)	(i) TES caused significant improvement in VA and VF (ii) TES reduced RP visual dysfunctions (iii) TES induced retinal blood flow. (iv) TES improved visual function (v) MVF regulated visual function	United States	Active, not recruiting	2014

NCT02405143	Stroke, infarction; posterior cerebral artery, hemianopsia	Active tACS using DC-stimulator MC	50 (18-75)	NA	Finland	Unknown status	2015

NCT02495935	Amblyopia	OkuStim®	40 (≥18)	NA	United States	Recruiting	2015

NCT02699216	Dry MD	ES of the retina	0 (≥50)	NA	NA	Withdrawn	2016

NCT03239418	Blepharoptosis Lagophthalmos	Neuromuscular ES	60 (≥19)	NA	United States	Unknown status	2017

NCT02548572	RP	Transcorneal ES using Okustim device	0 (22-80)	NA	United States	Withdrawn	2019

NCT04042363	MS, optic neuritis	Transorbital ES (eyetronic next wave 1.1)	45 (18-60)	NA	France	Recruiting	2019

NCT04010994	Glaucoma	Transorbital ES	50 (≥18)	NA	Germany	Enrolling by invitation	2019

NCT04017234	Myopia	Frequency following response, eye exercise, and transcutaneous	27 (6-13)	NA	Taiwan	Recruiting	2019

NCT02540148	Dry AMD	Nova oculus™ microcurrent ES	60 (≥50)	NA	Canada	Recruiting	2019

NCT04634383	Ocular injury Optic nerve diseases Photoreceptor degeneration Blindness, acquired	WFMA—wireless floating microelectrode array	5 (18-65)	NA	United States	Recruiting	2020

NCT04648085	Dry eye Neuropathic pain Eye pain	Transcutaneous ES	42 (>18)	NA	Mexico	Recruiting	2021

AMD: age-related macular degeneration; EEG: electroencephalogram; ES: electrical stimulation; *L*: location; MD: macular degeneration; ME: macula edema; MS: multiple sclerosis; *N*: number of patients; NAION: nonarteritic anterior ischemic optic neuropathy; NA: not available; POAG: primary open angle glaucoma; RP: retinitis pigmentosa; rtACS: repeated transcranial alternating current stimulation; VA: visual acuity; VF: visual field.
